# The data on the dispersion modeling of traffic-related PM_10_ and CO emissions using CALINE3; A case study in Tehran, Iran

**DOI:** 10.1016/j.dib.2018.07.019

**Published:** 2018-07-09

**Authors:** Mohammad Hadi Dehghani, Sima Jarahzadeh, Mostafa Hadei, Nabiollah Mansouri, Yousef Rashidi, Mahmood Yousefi

**Affiliations:** a Department of Environmental Health Engineering, School of Public Health, Tehran University of Medical Sciences, Tehran, Iran; b Institute for Environmental Research, Center for Solid Waste Research, Tehran University of Medical Sciences, Tehran, Iran; c Islamic Azad University, Science and Research Campus, Department of Environmental Engineering, Tehran, Iran

**Keywords:** Air pollution, CALINE3 model, Particulate matter, Carbon monoxide, Yadegar-e Emam Expressway

## Abstract

CALINE3 model predicts the dispersion of pollutants released from roadways in the receptor places at a certain radius from the source. This model was used to evaluate the dispersion of particulate matter < 2.5 µm (PM_10_) and carbon monoxide (CO) emitted from Yadegar-e-Emam Expressway (YEE) as one of the most congested highways in Tehran. The hourly concentrations of PM_10_ and CO, and the count and speed of vehicles were obtained from Tehran׳s Air Quality Control Company (TAQCC). Wind speed and direction, the height of mixing zone, air temperature, relative humidity, and stability class were acquired from IRAN Meteorological Organization (IRIMO). The emission factors (EF) of vehicles were acquired from those proposed for UK. The dispersion of PM_10_ and CO was predicted over the nearby area, and the modeled concentrations were estimated for a specific point, where an air quality monitoring station was working. The major portion of PM_10_ and CO released by vehicles in YEE was dispersed to the east. The comparison between the modeled and measured concentrations revealed that CALINE3 underestimates the concentrations of PM_10_ and CO by about 50%.

## Specifications Table

**Table:** 

Subject area	Environmental Health Science
More specific subject area	Air pollution
Type of data	Tables and Figures
How data was acquired	The hourly concentrations of PM_10_ and CO, and the count and speed of vehicles were obtained from Tehran׳s Air Quality Control Company (TAQCC). Wind speed and direction, the height of mixing zone, air temperature, relative humidity, and stability class were acquired from IRAN Meteorological Organization (IRIMO). The emission factors (EF) of vehicles were acquired from those proposed for UK.
Data format	Raw and analyzed
Experimental factors	The hourly, daily, weekly, monthly, seasonal, and annual averages of PM_10_ and CO were calculated and prepared for model.
Experimental features	The dispersion of PM_10_ and CO was predicted over the nearby area, and the modeled concentrations were estimated for a specific point, where an air quality monitoring station was working.
Data source location	Tehran, Iran.
Data accessibility	Data are included in this article

## Value of the data

• Urban transportation is one of the main causes of air pollution in cities [1]. This data article models the concentrations of PM_10_ and CO emitted from a highway in Tehran, which is a high-populated city with heavy traffic.
• This is the first study on the dispersion modelling of traffic-related air pollutants in Iran.
• The data in this study evaluates the performance of CALINE3 model to predict the dispersion of PM_10_ and CO.
• The data in this article indicates that CALINE3 underestimates the concentrations of PM_10_ and CO by about 50%.

## 1 Data

**Fig. 1:**
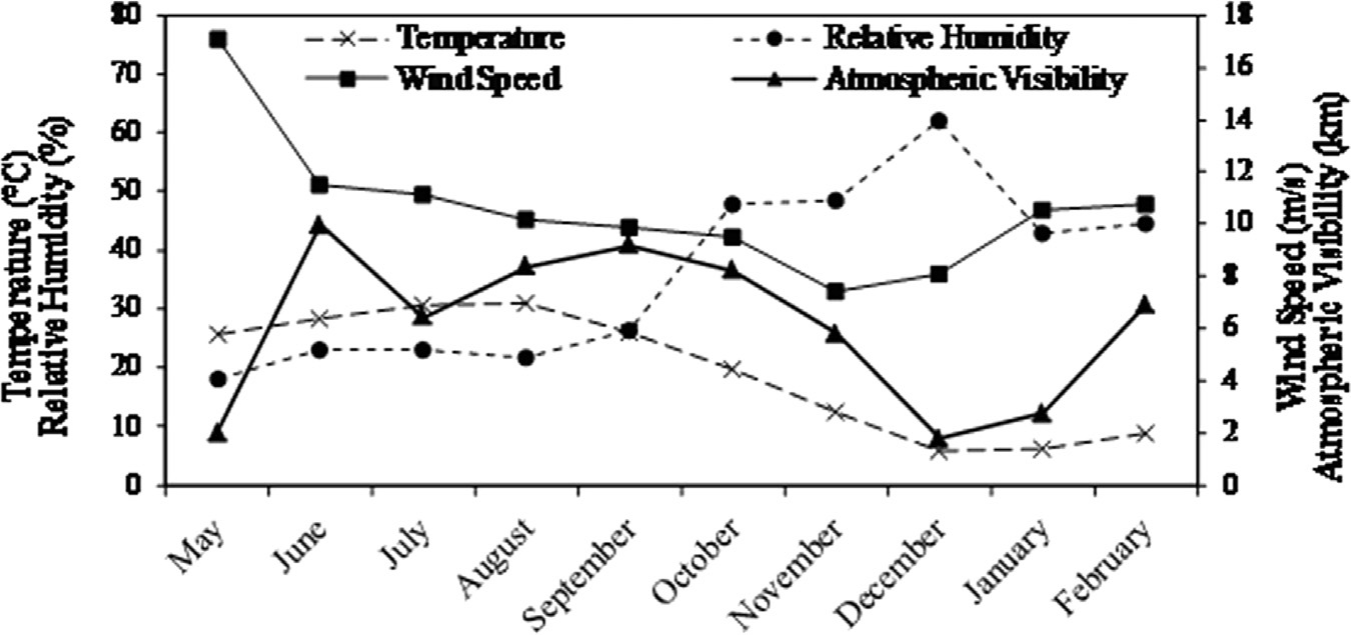
Monthly variations of wind speed, air temperature, relative humidity, and atmospheric visibility during 2011.

Fig. 1 illustrates the monthly variations of wind speed, air temperature, relative humidity, and atmospheric visibility during 2011. According to this Figure, the levels of wind speed, air temperature, and atmospheric visibility are lower during the winter; however, the relative humidity has increased within the same period.

**Fig. 2:**
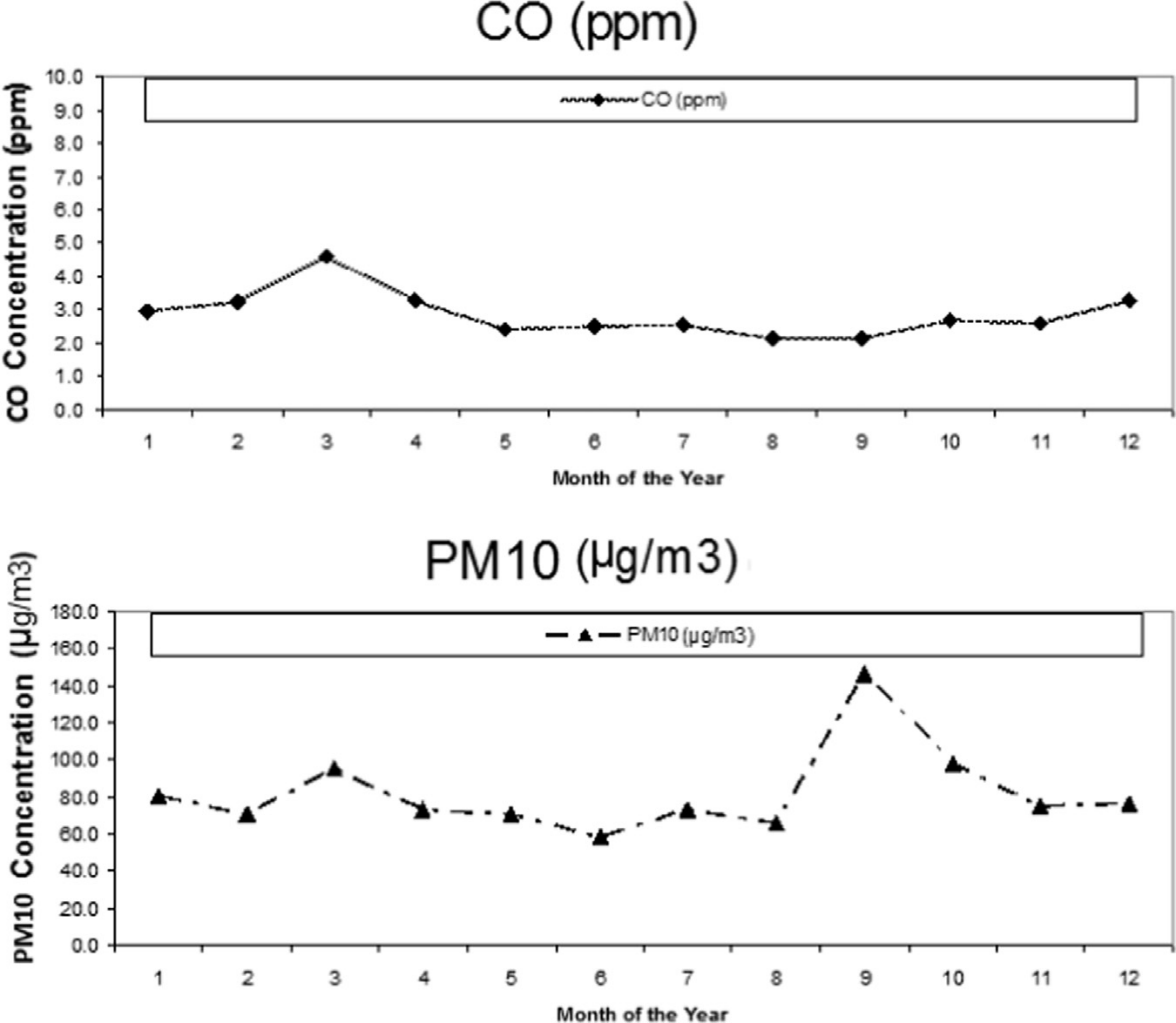
Monthly concentrations of carbon monoxide and PM_10_ during 2011.

Fig. 2 shows the hourly and monthly concentrations of carbon monoxide and PM_10_ during 2011, respectively. The highest concentrations of CO and PM_10_ were observed in warm and cold seasons, respectively.

**Fig. 3:**
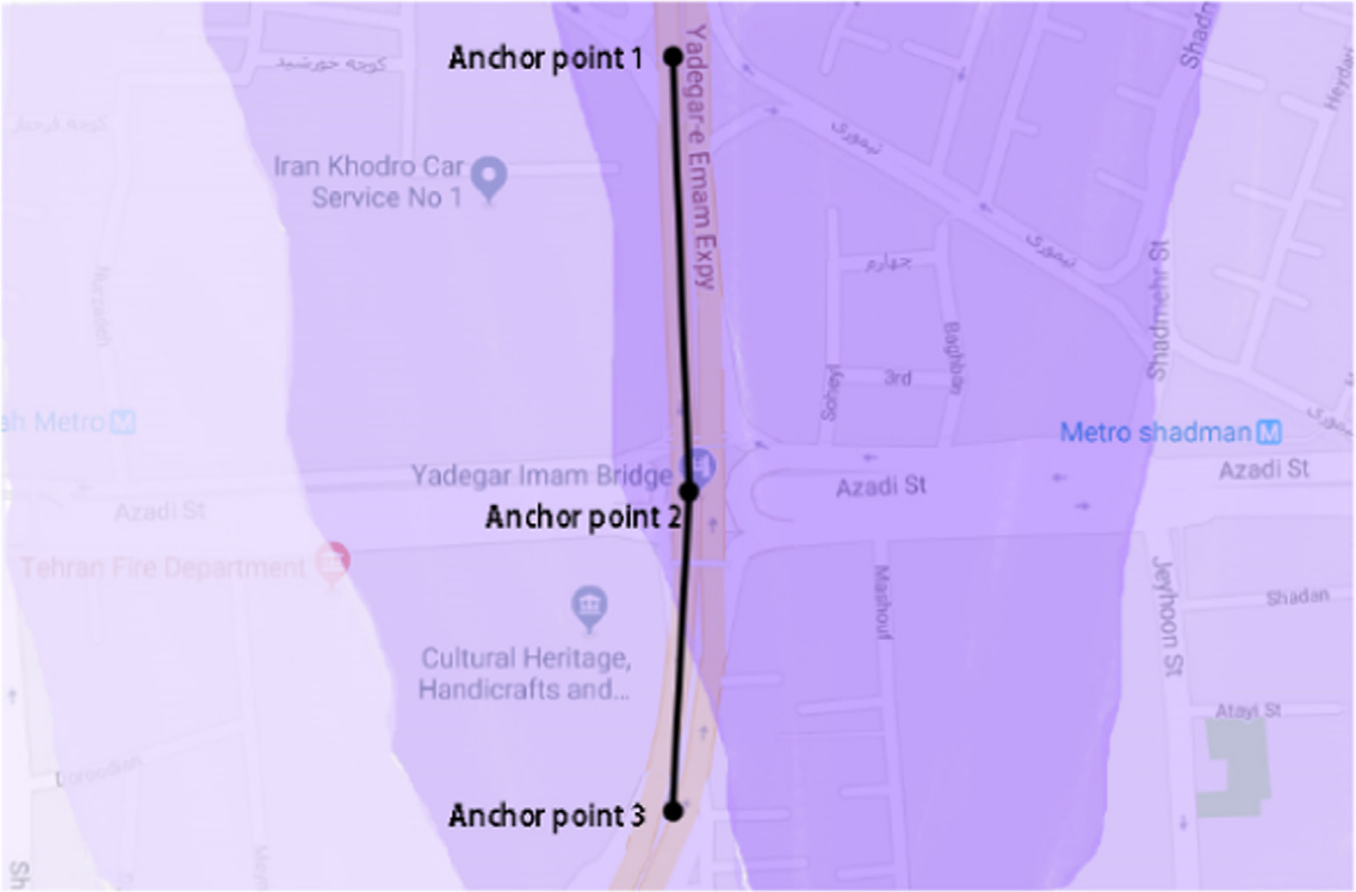
Modelled concentrations of carbon monoxide.

**Fig. 4:**
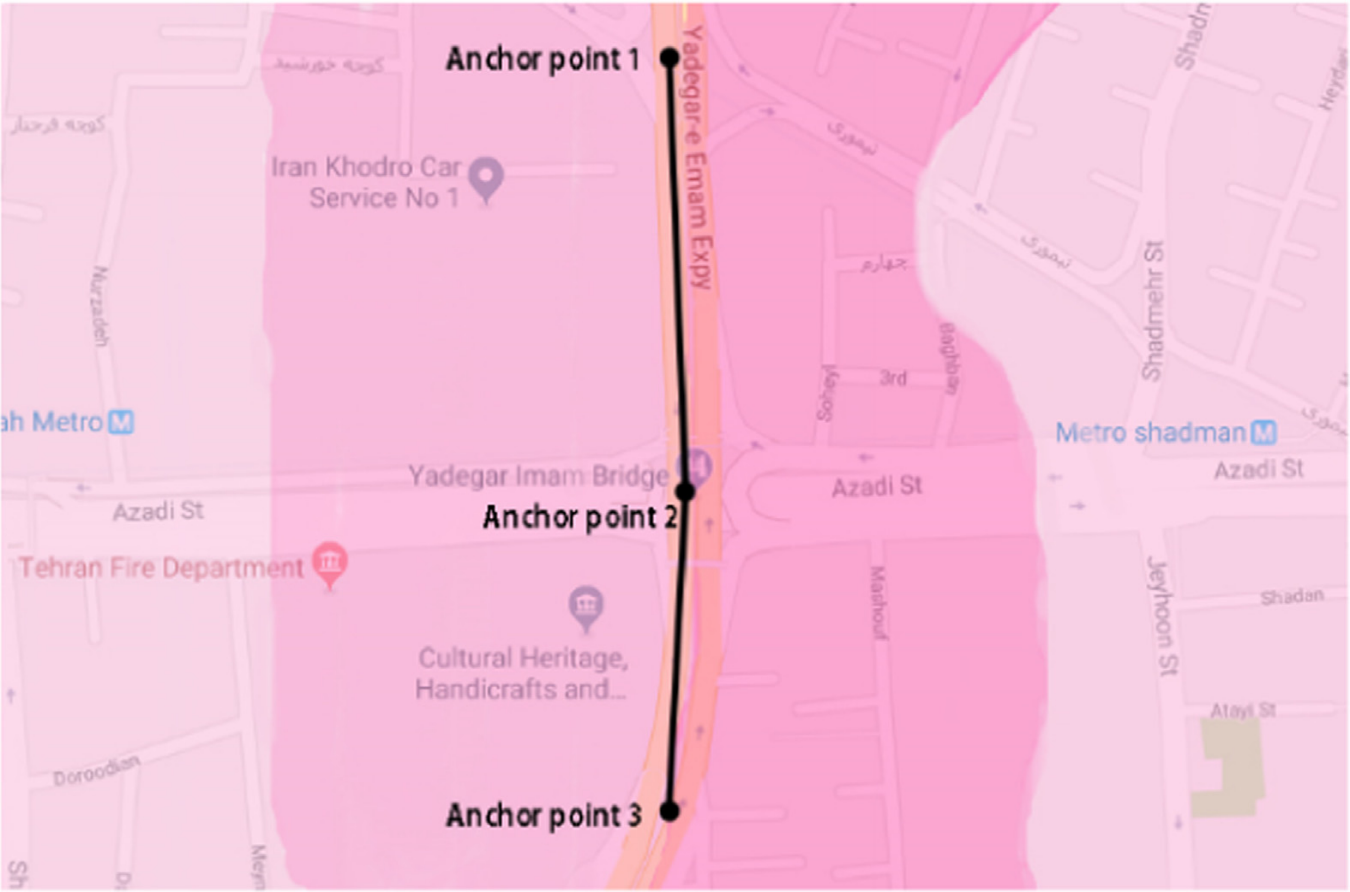
Modelled concentrations of PM_10_.

The modelled concentrations of carbon monoxide and PM_10_ are illustrated in Fig. 3, Fig. 4. These figures show the dispersion of these pollutants emitted from the highway in the nearby area. The comparison between the modeled and measured concentrations revealed that CALINE3 underestimates the concentrations of PM_10_ and CO by about 50%.

## 2 Experimental design, materials and methods

### 2.1 Study area and period

This study was conducted in Tehran during the 2011. CALINE3 model was used to evaluate the dispersion of PM_10_ and CO emitted from Yadegar-e-Emam Expressway (YEE) as one of the most congested highways in Tehran. This highway is at the intersection of Azadi Street, which is a main street in Central areas of Tehran.

### 2.2 CALINE3

This model characterizes pollutant dispersion over the roadways. CALINE3 divides an individual road/highway link into a series of elements to calculate the incremental concentrations. Afterward, it aggregates all elements to estimate the total concentration at a specific receptor place [2], [3]. Basic equation of CALINE3 is as follows:

(1) $$C\left(x,y\right)=\frac{q}{\pi \sigma_{z}u}\int_{y_{1}-y}^{y_{2}-y}\exp\left(-\frac{y^{2}}{2\sigma_{y}^{2}}\right)\mathrm{dy}$$

where, *q*, *u*, *σ_y_* and *σ_z_* are the linear source strength (g/m s), the wind speed (m s^−1^), the horizontal and vertical diffusion coefficients (m), respectively. Additionally, *x*, *y*, *y*_1_ and *y*_2_ denote to the downwind distance of the receptor (m), the crosswind distance of the receptor (m), and the endpoint y-coordinates of the elements (m), respectively.

CALINE3 calculates the linear source strength considering constant emissions all over the element. The first element considers the area directly above the road as a zone of constant emission and turbulence, known as the mixing zone, which is a region above the interest road plus 3 m from each side. This further width in each side accounts for an initial horizontal dispersion by the vehicle wake effect. The model incorporates the wind angles in terms of azimuth bearings. The important feature of this model is that it accounts for the increased emission factors for the vehicles approaching very close to the intersection [2], [3].

### 2.3 Data requirements

**Fig. 5:**
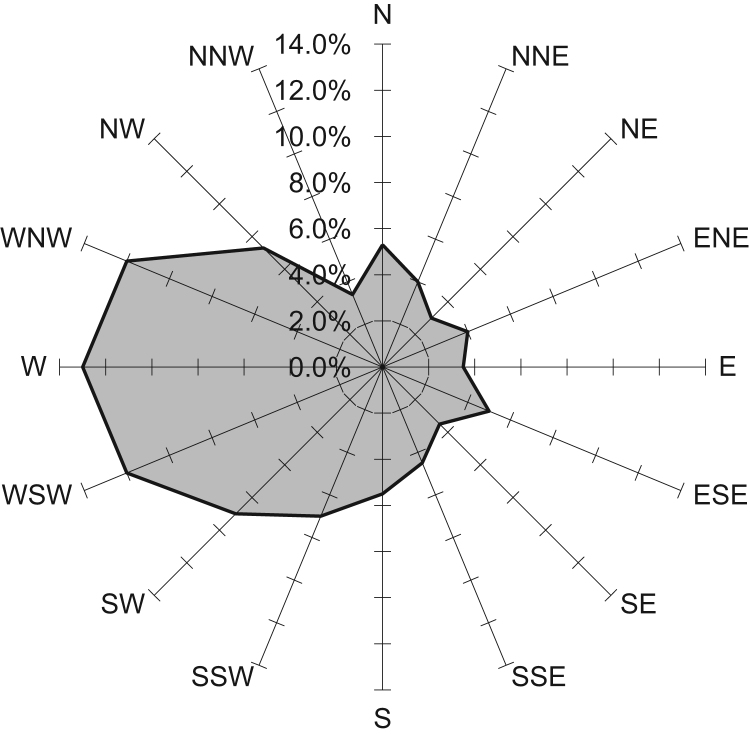
Direction and frequency of winds in Tehran.

The hourly concentrations of PM_10_ and CO were obtained from Tehran׳s Air Quality Control Company (TAQCC). The hourly, daily, weekly, monthly, seasonal, and annual averages of PM_10_ and CO were calculated and prepared for model. The meteorological data such as wind speed and direction, the height of mixing zone, air temperature, relative humidity, and stability class were acquired from IRAN Meteorological Organization (IRIMO). The rose wind used in this data article is presented in Fig. 5.

Traffic data included the count and speed of vehicles and their emission factors per each vehicle. The data about the vehicles were obtained from TAQCC. Due to lack of local data about the emission factors (EF) of Iranian vehicles, the values were acquired from those proposed for UK [4]. In this case, transportation fleet in Tehran were divided to 4 groups: light-duty gasoline cars, buses, motorcycles, and diesel-duty vehicles. Considering an average speed of 80 km/h and Euro II emission standards, emission factors for free-flowing conditions used in this data article are presented in Table 1. The emission factors were further combined to calculate the ultimate EF to provide inputs to the source strength of models. The ultimate EF was obtained using the following equation [5]:

(2) $${\mathrm{EF}}_{c}=\frac{{\mathrm{EF}}_{1}\times V_{1}+{\mathrm{EF}}_{2}\times V_{2}+{\mathrm{EF}}_{3}\times V_{3}+{\mathrm{EF}}_{4}\times V_{4}}{\Sigma V}$$

where, *V*_1_–*V*_4_ are Petrol-fueled personal vehicles, buses, motorcycles, and Diesel-fueled vehicles, respectively. *EF*_1_–*EF*_4_ are the emission factors corresponding to the same groups of vehicles, respectively.

**Table 1:** Emission factors of four groups of vehicles (*V* = 80 km/h, emission standard = Euro II).

**Groups**	**PM**_**10**_**(g/km)**	**CO (g km)**
Petrol-fueled personal vehicle	0.0053	0.607
Bus	0.120	0.580
Motorcycle	0.0047	0.580
Diesel-fueled vehicles	0.063	0.073

### 2.4 Modelling

A five hundred meter section of Yadegar-e-Emam Expressway (YEE) was chosen to enter the model. This straight section of YEE was divided into 2 sub-links using 3 points. The modelling was performed for each of these 2 sub-links. The required data were entered to the CALINE3, and the dispersion of YEE-emitted PM_10_ and CO was estimated, and compared to those measured by a nearby monitoring station.
